# Development of an advanced in-line multilayer deposition system at Diamond Light Source

**DOI:** 10.1107/S1600577524006854

**Published:** 2024-08-09

**Authors:** Hongchang Wang, Arindam Majhi, Wai Jue Tan, Wadwan Singhapong, Christian Morawe, Kawal Sawhney

**Affiliations:** a Diamond Light Source Didcot United Kingdom; b Department of Mechanical Engineering University of Bath Bath United Kingdom; c European Synchrotron Radiation Facility Grenoble France; DESY, Germany

**Keywords:** multilayers, multilayer deposition system, synchrotron radiation, optics, Diamond-II

## Abstract

A state-of-the-art multilayer deposition system with a 4200 mm-long linear substrate translator housed within an ultra-high vacuum chamber has been developed. This instrument is engineered to produce single and multilayer coatings, accommodating mirrors up to 2000 mm in length through the utilization of eight rectangular cathodes. To ensure the quality and reliability of the coatings, the system incorporates various diagnostic tools for *in situ* thickness uniformity and stress measurement. Furthermore, the system features an annealing process capable of heating up to 700°C within the load-lock chamber. The entire operation, including pump down, deposition and venting processes, is automated through user-friendly software. In addition, all essential log data, power of sputtering source, working pressure and motion positions are automatically stored for comprehensive data analysis. Preliminary commissioning results demonstrate excellent lateral film thickness uniformity, achieving 0.26% along the translation direction over 1500 mm in dynamic mode. The multilayer deposition system is poised for use in fabricating periodic, lateral-graded and depth-graded multilayers, specifically catering to the beamlines for diverse scientific applications at Diamond Light Source.

## Abstract 2

A state-of-the-art multilayer deposition system has been developed at Diamond Light Source; this instrument is engineered to produce single and multilayer coatings, accommodating mirrors up to 2000 mm in length through the utilization of eight rectangular cathodes.

## 1. Introduction

Multilayer (ML) optics for X-rays employ a stratified structure comprising alternating layers of high and low atomic number materials. Treated as synthetic crystals with adaptable *d*-spacing, these optics effectively cover a broad energy spectrum. In synchrotron facilities, their versatility propels advancements in the field across various applications, including ML monochromators, gratings, nano-focusing devices, polarizing elements and power filters (Spiller, 1998 ▸; Attwood, 1999 ▸; Bajt *et al.*, 2007 ▸; Macrander & Huang, 2017 ▸; Lider, 2019 ▸). Notably, ML optics yield significantly higher photon flux (Δ*E*/*E* = 1–2%) compared with natural crystal (Δ*E*/*E* of 0.01%). Furthermore, high-resolution X-ray MLs (Δ*E*/*E* = 0.2–0.4%) have been further developed to bridge the gap between conventional MLs and crystal optics (Morawe *et al.*, 2001*a* ▸,*b* ▸; Morawe, 2019 ▸). An exceptional feature of ML optics lies in their ability to achieve around 10 nm focusing for hard X-rays, showcased prominently by ML-coated curved mirrors and ML Laue lenses (MLLs) (Kang *et al.*, 2006 ▸, 2008 ▸; Yan *et al.*, 2013 ▸; Conley *et al.*, 2009 ▸; Morawe *et al.*, 2015 ▸; Mimura *et al.*, 2011 ▸; Bajt *et al.*, 2018 ▸; Dresselhaus *et al.*, 2024 ▸). In addition, the conventional crystal or single layer coated grating based monochromators do not work efficiently in the tender X-ray energy range, yet ML gratings have the potential to increase the diffraction efficiency of monochromators by up to one order of magnitude (Yang *et al.*, 2017 ▸; Sokolov *et al.*, 2019 ▸). Moreover, ML polarizing elements stand out as indispensable tools, facilitating complete polarization analysis of light in the soft X-ray region (Wang *et al.*, 2012 ▸; Zhu *et al.*, 2020 ▸; Schäfers *et al.*, 1999 ▸).

Advancements in X-ray optics based on MLs have been made possible over recent decades through the development of various deposition systems (Störmer *et al.*, 2011 ▸; Ni *et al.*, 2019 ▸; Morawe *et al.*, 2022 ▸; Conley *et al.*, 2014 ▸). The magnetron sputter deposition method stands out as the preferred approach for depositing MLs due to its ability to produce films that are denser and smoother and it provides higher kinetic energy, stability, precise thickness control and repeatability (Kazimirov *et al.*, 2006 ▸; Martynov *et al.*, 2004 ▸; Nayak *et al.*, 2015 ▸; Wei *et al.*, 2022 ▸). In this technique, the combination of electric and magnetic fields is utilized to bombard a target material with ions, resulting in the sputtering of atoms that are then deposited as a thin film onto a substrate. Particularly well suited for optics with a modest footprint, the conventional rotary deposition system facilitates high-speed substrate rotation, ensuring highly uniform film deposition.

However, long and narrow ML optics with a cuboid shape are extensively employed in synchrotron sources, because of the smaller beam footprint and grazing angle of incidence of the X-ray beam. For example, the length of an ML monochromator mirror varies from 150 mm up to 900 mm (Morawe *et al.*, 2017 ▸). Additionally, there is an instance of a 1000 mm-long focusing ML mirror, employed to capture and manipulate large beams emitted from wiggler sources (Sutter *et al.*, 2019 ▸). The growing demand for large X-ray mirrors has prompted the establishment of linear deposition systems. The distinctive advantage of these facilities is that they can achieve excellent lateral uniformity by moving the mirror at a constant speed. In addition, the custom-designed thickness profile can be deposited by adjusting the substrate motion speed during a deposition process. These linear deposition facilities are capable of accommodating mirror substrates ranging widely in length, from 300 mm to 1500 mm (Conley *et al.*, 2014 ▸; Morawe *et al.*, 2022 ▸; Ni *et al.*, 2019 ▸; Störmer *et al.*, 2008 ▸). These advancements represent a significant stride in meeting the evolving technological demands associated with a wide range of X-ray ML optics in synchrotron applications.

At Diamond Light Source (DLS), ML optics will be used across multiple beamlines serving as ML monochromators (Sawhney *et al.*, 2011 ▸; Sutter *et al.*, 2019 ▸), soft-X-ray polarimeters (Wang *et al.*, 2011 ▸, 2012 ▸) and ML gratings (Yang *et al.*, 2017 ▸). With the forthcoming upgrade to Diamond-II, the demand for high-quality X-ray mirrors has risen to facilitate nano-focusing and enhance the efficiency of tender X-ray beams at several beamlines. Procuring large MLs with specialized geometries is challenging as some are not commercially available and cannot be fabricated by conventional deposition systems. Consequently, at the DLS, an in-line multilayer deposition system (MDS) has been developed by Bestec GmbH (Germany) with a design concept similar to that of the ESRF compact ML coating system (Morawe *et al.*, 2022 ▸). This MDS can accommodate mirror substrates up to 2000 mm in length. In this paper, we present the key features of the system including capabilities for *in situ* thickness uniformity characterization, stress measurement, and annealing and cleaning processes. This advanced MDS will produce a wide range of custom-designed and specialized ML optics suitable for numerous beamlines across various applications.

## 2. Multilayer deposition system

**Figure 1:**
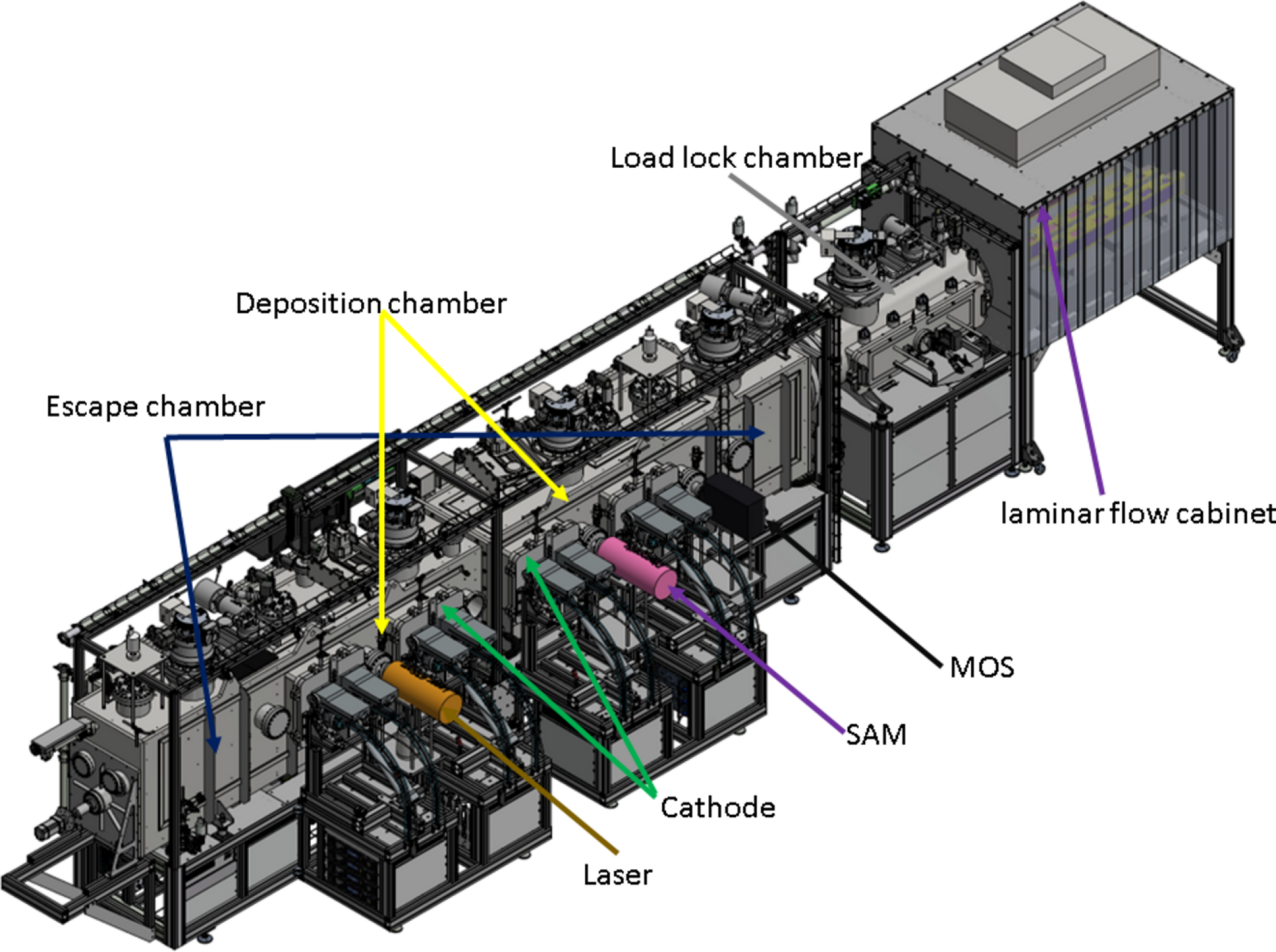
Schematic layout of the MDS at DLS.

A recently installed and state-of-the-art MDS utilizes direct current (DC) magnetron sputter deposition for monolayer or ML fabrication. It is configured as an in-line system, featuring a deposition chamber, two escape chambers and a separately pumped load-lock chamber. At the centre of the system lies a deposition chamber with sputter sources and associated equipment. Adjacent to it are two escape chambers, facilitating horizontal movement of substrates in front of the sputter sources. Both the cathodes and the optical surface are vertically oriented, situated opposite each other within the system. The MDS accommodates a total of eight cathodes along the motion direction of the carrier. Each cathode, measuring 254 mm × 89 mm, is designed to ensure optimal flux uniformity. Each cathode is equipped with its individual fast shutter mechanism to enable rapid opening or blocking of particle flux. Customer-designed masks can be integrated to tailor particle flux distribution. Additionally, the distance between the cathodes and the substrate surface is manually adjustable from 50 mm to 100 mm. For ease of access during modifications, maintenance and repair, the MDS is equipped with large service doors. A schematic setup of the MDS is presented in Fig. 1 ▸, offering a comprehensive overview of its functional components and layout.

The substrate carrier is designed to accommodate substrates with diverse sizes. This carrier with substrates less than 1000 mm long can be loaded into the load-lock chamber. It also allows for the installation of glass substrates without obstructing the passage of a laser beam, essential for precise thickness-uniformity measurements. The primary working gas for the system is argon, though the flexibility to use alternative gases such as oxygen or nitro­gen is also incorporated. The cathodes can reliably ignite and operate at working pressures of 1 × 10^−3^ mbar. To ensure control of process gas in the upstream mode, each cathode is equipped with an individual gas inlet and flow meter. Meanwhile, in the downstream mode, the system operates in a constant pressure mode, utilizing constant inlet gas flow and variable pumping speed through throttle valves.

The load-lock chamber is connected to the deposition chamber via a gate valve. This chamber facilitates sample transfer to the deposition chamber without venting the whole system. Additionally, the load-lock chamber boasts the inclusion of four ultraviolet lamps, instrumental in removing carbon contamination from mirror surfaces and pre-processing substrate surfaces. Further enhancing its capabilities, a heating device is provided for annealing small substrate surfaces (25 mm × 25 mm) up to temperatures of approximately 700°C. A laminar flow clean bench is integrated with the load-lock chamber, and this bench establishes an aseptic space in the loading area, effectively preventing contamination from dust when loading X-ray mirror substrates into the load-lock chamber.

For vacuum monitoring, two wide-range vacuum gauges are positioned in both the load-lock and the deposition chambers. One gauge oversees venting pressure control, while the other manages foreline pressure. To ensure the precise control of process gas pressure during deposition, two dedicated absolute pressure gauges are mounted in the deposition chamber near the sputter sources. The mounting design of these pressure gauges is crafted to be protected against vibrations and thermal drifts, utilizing small edge-welded bellows to connect to the deposition and etching vacuum chamber, effectively minimizing vibrations (Conley *et al.*, 2012 ▸).

**Figure 2:**
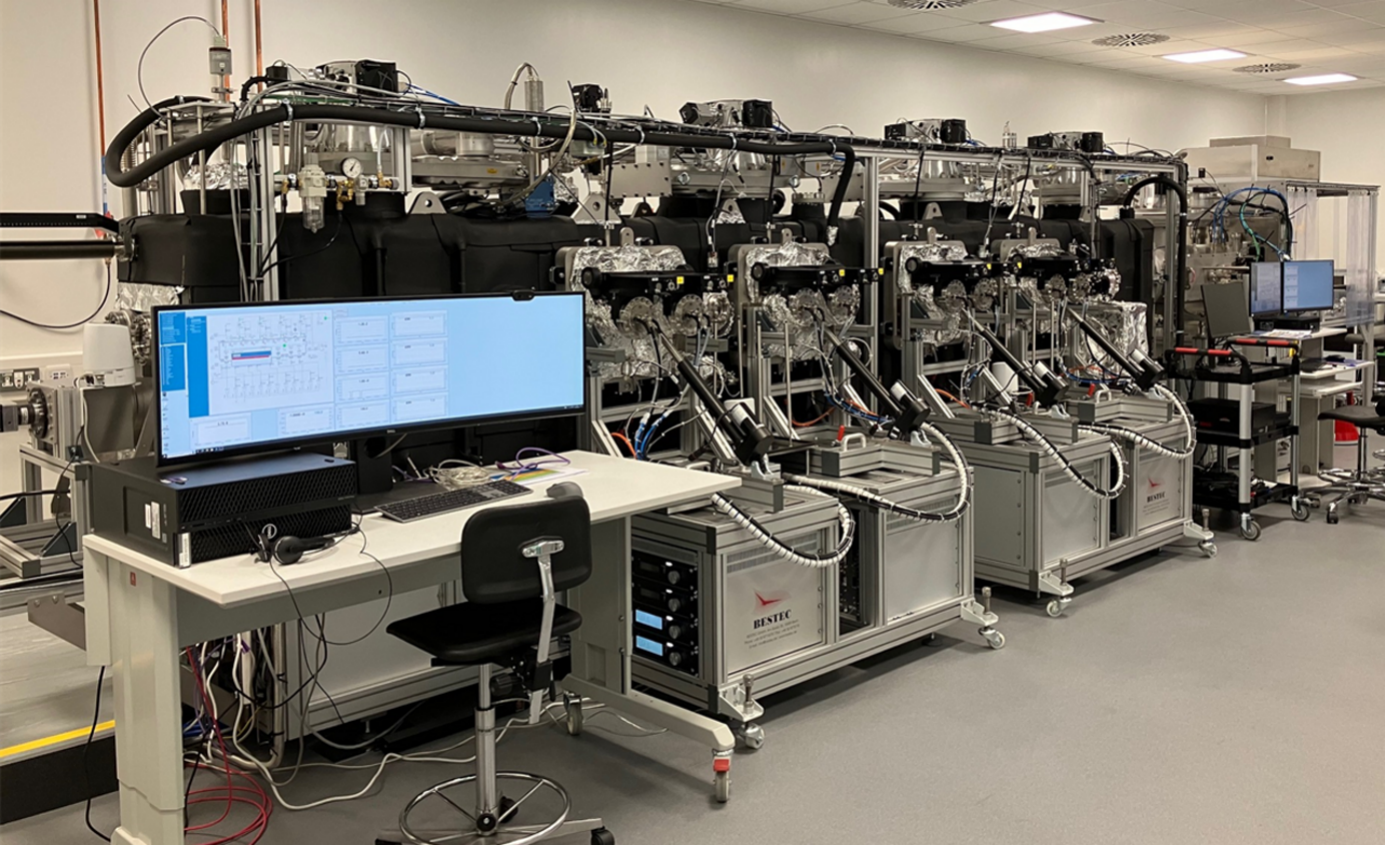
Photograph of the MDS at DLS.

The deposition chamber features a combination of dry primary pumps and four symmetrically mounted turbo-molecular high-vacuum pumps. A base pressure of 3 × 10^−8^ mbar was achieved within one week without requiring a bake-out process. Additionally, the system is enhanced with both bake-out lamps and wires, effectively heating the chamber up to 150°C, expediting the pumping process. The load-lock chamber is pumped down within 4 h, reaching a pressure of about 10^−7^ mbar. Two types of DC power supplies are employed to energize the sputter sources, catering to both high-resolution and high-power modes, boasting a capacity of up to 1500 W. This comprehensive configuration ensures not only optimal performance but also precise control of the MDS (see Fig. 2 ▸). The functional specification of the MDS is provided in Table 1 ▸.

The in-vacuum carrier motion system is a critical component in the machine design. The in-vacuum carrier motion system has a total stroke of 4200 mm, accounting for the width of the deposition zone and providing ample space for acceleration. The design of the MDS is to achieve a uniform or user-defined thickness profile on a long substrate, utilizing four centrally positioned sputter cathodes within the deposition chamber. In theory, the MDS is capable of deposition of an ML mirror with lengths up to 2000 mm and a single-layer coating up to 2400 mm.

The carrier/motion system can ensure that the substrate surface is positioned precisely with a travel speed between 0.1 mm s^−1^ and 100 mm s^−1^. The longitudinal positioning accuracy of the substrate is within ±0.1 mm along the full length of the carrier motion. The speed accuracy is ±0.1% within the speed range from 10 mm s^−1^ to 50 mm s^−1^. To achieve and measure the above performance requirements, the motion system is equipped with a Renishaw Resolute absolute encoder that directly measures the instantaneous carriage position.

An industrial PC based control system has been developed to control the pump systems, gas flow, pressure control, valve and shutter control, generator and power supply, and motion control. Users can program their own macro scripts for different deposition processes. All essential log data, power of sputtering source, working pressure and motion positions are displayed *in situ* during the deposition process and automatically stored for comprehensive data analysis.

In addition, we have allocated spare viewports, reserved to facilitate the integration of forthcoming updates, thereby ensuring our system remains poised for continuous advancements. Notably, an ion source is envisaged for deterministic figuring of the X-ray mirror (Conley *et al.*, 2014 ▸; Idir *et al.*, 2015 ▸), and a plasma source can be installed for *in situ* cleaning of X-ray mirrors before the coating process. The implemented speckle angular measurement instrument can be used in onboard metrology for the X-ray mirrors (Wang *et al.*, 2021 ▸). Furthermore, the utilization of an eight-target configuration can be potentially used for upcoming developments in MLL (Conley *et al.*, 2009 ▸).

## 3. *In situ* diagnostic system

**Figure 3:**
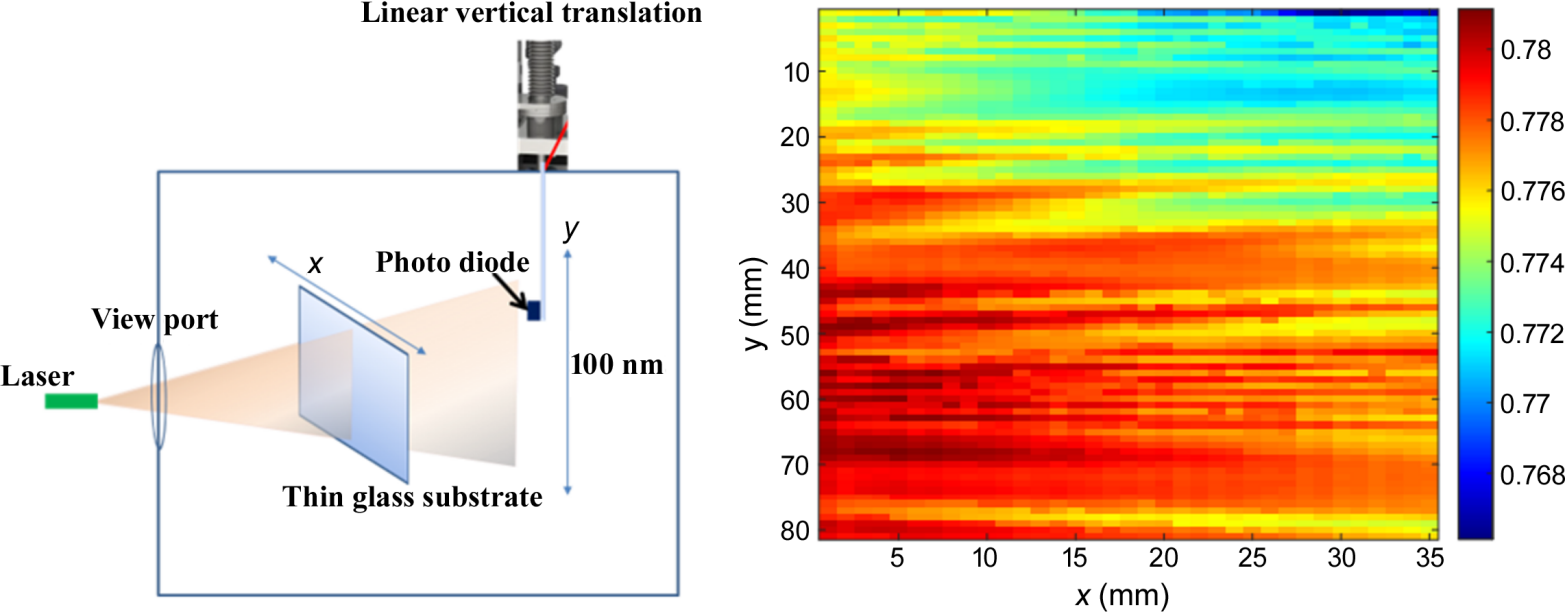
(Left) Schematics of the *in situ* thickness-uniformity measurements inside of the sputtering chamber. (Right) Measured transmission intensity profile with a laser and photodiode for one sample.

In comparison with the conventional static mode, where samples remain fixed during deposition, the dynamic mode offers superior uniformity along the mirror translation direction by continuously moving the sample across the sputtering source at a constant speed. A figured mask can be employed to further enhance the thickness uniformity along the transverse direction (Conley *et al.*, 2008 ▸, 2007 ▸, 2014 ▸; Spiller *et al.*, 1998 ▸, 1992 ▸; Montcalm *et al.*, 2001 ▸; Bajt *et al.*, 2008 ▸). However, the optimization of the mask shape can be time-consuming, particularly when measuring thickness profiles using X-ray diffractometry. To speed up thickness uniformity measurements, a laser-based transmission profile method has been developed. Here, a photodiode system has been integrated into the deposition chamber to enable *in situ* thickness-uniformity measurements. A schematic of the setup is given in Fig. 3 ▸ (left). A divergent laser is mounted on a platform next to the cathode and positioned to shine through the viewport window, traversing thin films coated on glass substrates. The transmitted light intensity is measured by a photodiode mounted on a vertical motorized translation stage. In its retracted position, the photodiode is shielded behind a plate to prevent contamination. The system allows for a raster scan along both the horizontal and the vertical directions before and after thin-film coating, enabling the recording of the laser intensity profile. Multiple glass samples can be accommodated on a large sample holder, facilitating the measurement of thickness distribution under various masking conditions. This integrated approach not only optimizes the dynamic deposition process, but also offers real-time monitoring and measurement of thickness uniformity within the deposition chamber. One piece of glass with thin-film coating has been measured with this method, and the measured transmission intensity profile is shown in Fig. 3 ▸ (right). It can be seen that the intensity profile is not uniform along the vertical direction of the mirror.

For real-time stress measurements of coated films, a multi-beam optical sensor (MOS) has been positioned on an angled viewport. The MOS employs a single laser to generate a 2D laser array of spots, reflecting them off the sample surface and onto a high-resolution detector. The principle of the MOS system can be described as follows: on a flat surface, the reflected beams maintain their original spacing, whereas on a curved surface, deflection occurs, altering the spacing on the detector. The MOS, by simultaneously measuring the spacing between laser spots in two orthogonal directions, provides comprehensive 2D curvature data. The stress measurement process involves monitoring substrate curvature with an MOS system. The MOS controller integrates with the system, supporting triggering signals and accepting signals generated by MDS software. The viewport window has been protected with a shutter to avoid contamination during the deposition, and the shutter will only open once the sample is moved to the front of the MOS.

The change in curvature (κ − κ_0_) is related to the film stress (σ) by the Stoney equation:
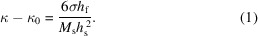
The other parameters in this equation are the film thickness (*h*_f_), biaxial modulus of the substrate (*M*_s_) and the substrate thickness (*h*_s_). The radius of curvature, *R*, is equal to 1/κ.

**Figure 4:**
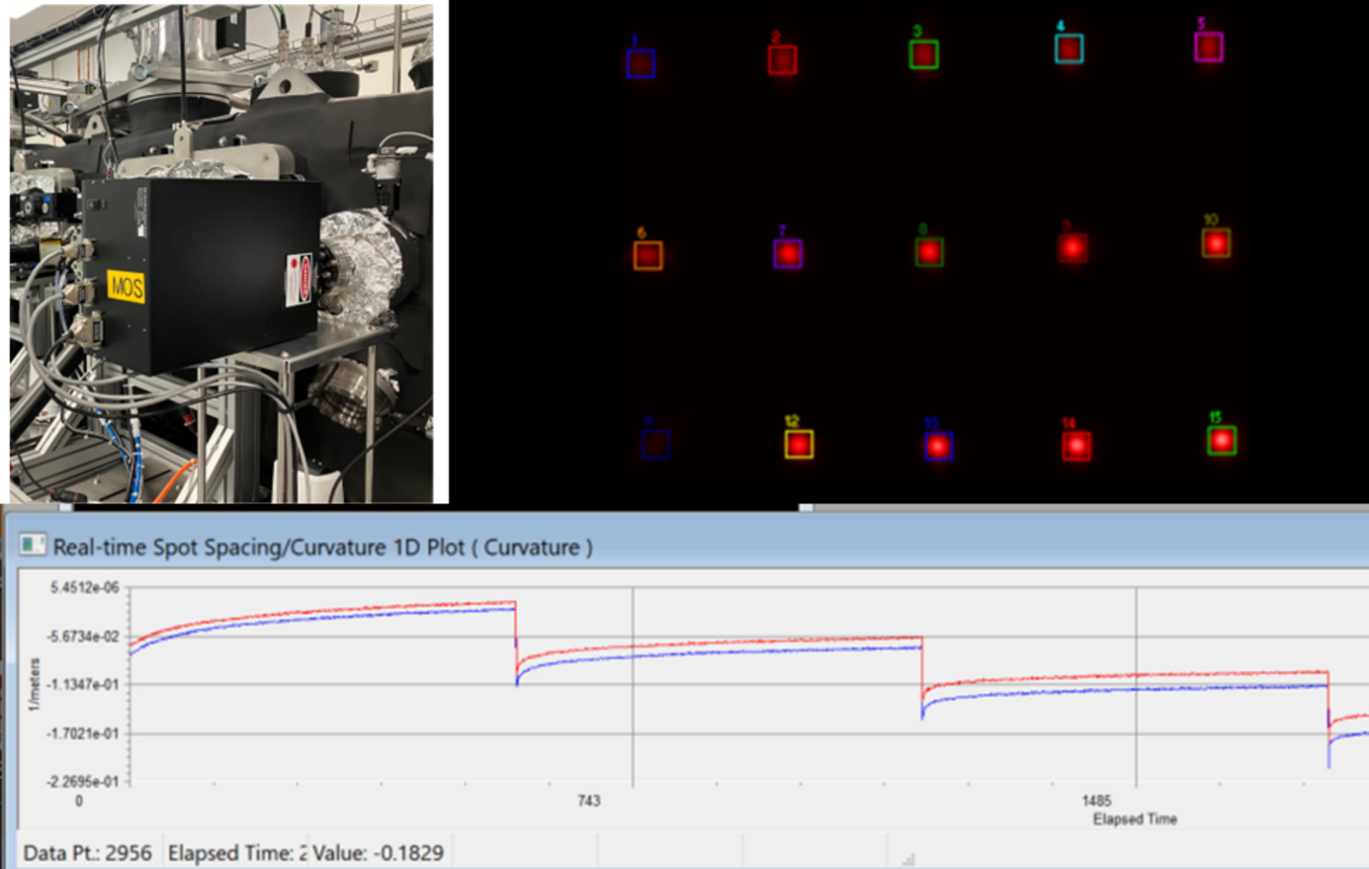
(Top left) Photograph of MOS installed on the deposition chamber. (Top right) Laser array collected by the camera. (Bottom) Measured curvature change along both the horizontal and the vertical directions by changing the voltage of the piezo actuator of a deformable mirror.

To demonstrate the capabilities of the MOS, a piezoelectric deformable mirror with a Ø10 mm pupil, suspended by biomorph benders, is placed in front of the MOS. The voltage across all 40 actuators underwent incremental changes of 10 V every 600 s. As illustrated in Fig. 4 ▸, the radius curvatures in both directions decreased with the adjustments in the actuator voltages. Note that the onset of voltage changes revealed hysteresis effects from the piezo actuator. Anticipating further tests with real thin films in future commissioning work underscores the potential of MOS for conducting comprehensive stress measurements.

## 4. Preliminary commissioning results

Once the MDS has been installed, a series of single-layer and ML coatings are fabricated using both static mode (where the substrate remains stationary in front of the cathode) and dynamic mode (where the substrate moves at either a constant or a variable speed) to explore the performance in the MDS system. The characterization of the X-ray optical performance of a single layer or an ML is performed by means of X-ray reflectivity (XRR) at Cu *K*α (λ = 0.15406 nm, *E* = 8048 eV) using a D8 Advance, Bruker AXS diffractometer in θ–2θ geometry. The reflectivity performance is carried out with an incident beam size of 0.1 mm and a reflected beam size of 0.6 mm. The reflectivity profiles are analysed with the *IMD* software (Windt, 1998 ▸). The optical constants used are taken from the Henke database. The fitting of an ML structure is accomplished by employing a two-layer model with adjustments made to the mass density, layer thickness and roughness in the overall stack. The periodic thickness *d* is calculated using the modified Bragg equation (Attwood, 1999 ▸, Macrander & Huang, 2017 ▸):
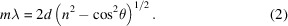
Here, λ is the wavelength, *m* is the number of Bragg diffraction order, *n* is the real part of refraction index and θ is the grazing incidence angle of the X-rays.

The initial commissioning results focus on two material combinations: Cr/C and W/B_4_C. The Cr/C ML grating with constant periodic thickness is developed for the B07 beamline plane grating monochromator operating in the spectral range 0.5 keV to 3 keV. The ML grating is optimized to have a 50 Cr/C bi-layer and an ML period of 10.2 nm. The Cr/C ML will be deposited on a laminar grating with a line density of 1200 lines mm^−1^ and a groove depth of 7.5 nm. The W/B_4_C lateral-graded ML is used for the polarimeter on the I21 beamline. The necessary parameters for the graded ML are as follows: W/B_4_C ML, with *N* = 100 and a period ranging from 1.88 nm to 2.00 nm with a clear aperture of 24 mm × 70 mm.

**Figure 5:**
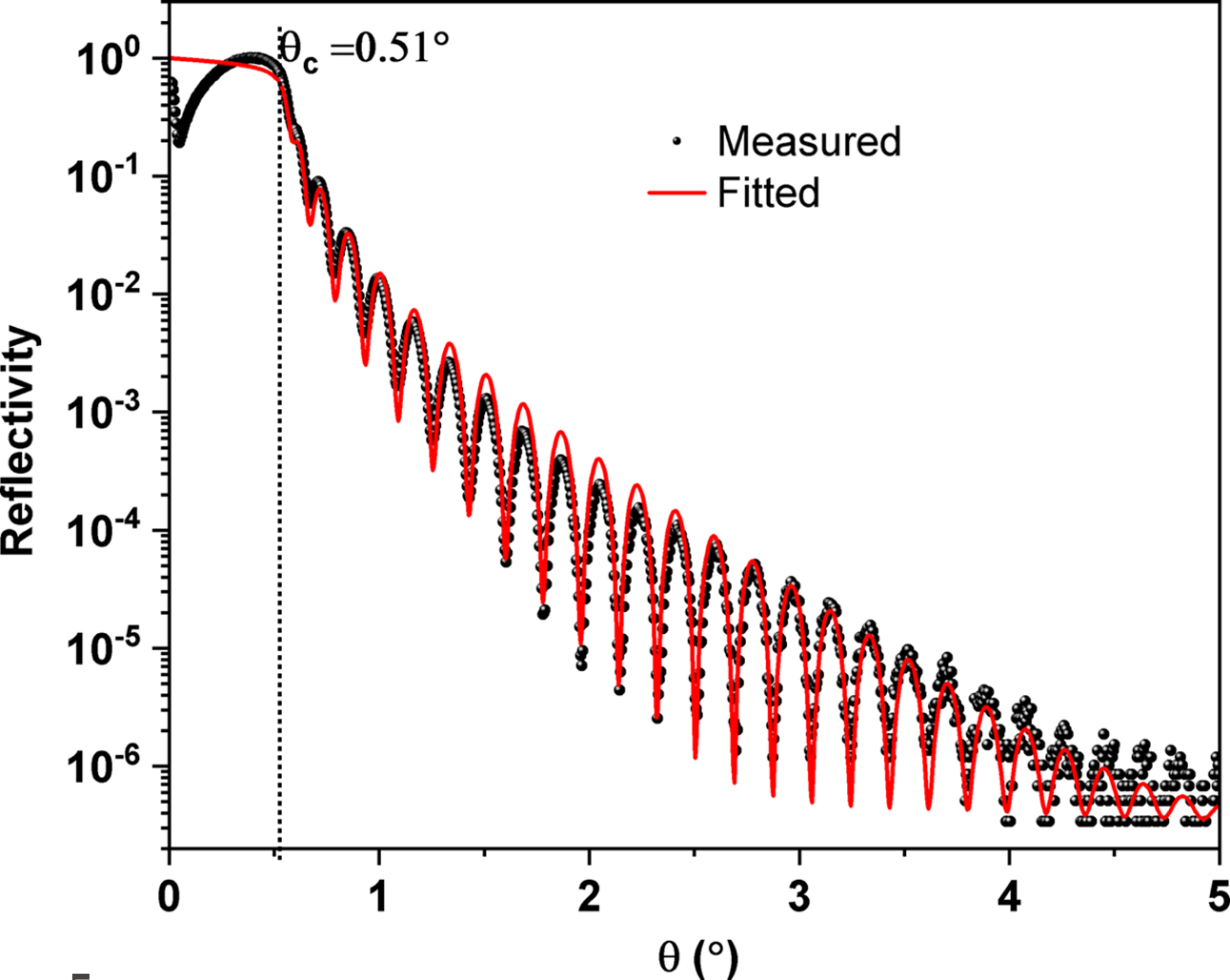
Measured XRR (black bubbles) and fitted profile (solid red line) of a W single layer of 23.48 nm thickness, 19.3 g cm^−3^ density and 0.36 nm roughness. The XRR is performed at *E* = 8048 eV.

A single-layer film of W is fabricated on an Si substrate using the following deposition parameters: working argon gas pressure of 3.2 × 10^−3^ mbar, substrate-to-target distance of 81 mm, cathode power of 100 W and a deposition time of 100 s in static mode. Fig. 5 ▸ illustrates the measured and fitted XRR of the fabricated W layer as a function of the incidence angle. As expected for a single layer, Kiessig fringes are clearly visible due to interference of the X-ray beams reflected at both interfaces (*i.e.* the Si/W and W/air interfaces). The critical angle (θ_c_) of this high-*Z* material amounted to 0.51°. The reflectivity curve is measured over six orders of magnitude. The maxima and minima of the reflectivity scan are clearly visible due to the high-density contrast between Si and W. The contrast is distinctly weaker for the other low-density materials such as carbon and boron carbide. The W layer thickness is approximately 23.48 nm, the film/interface roughness amounted to 0.36 nm and the layer density is fixed with a bulk density of 19.3 g cm^−3^. The calculated deposition rate of W in static mode was found to be 0.235 nm s^−1^.

**Figure 6:**
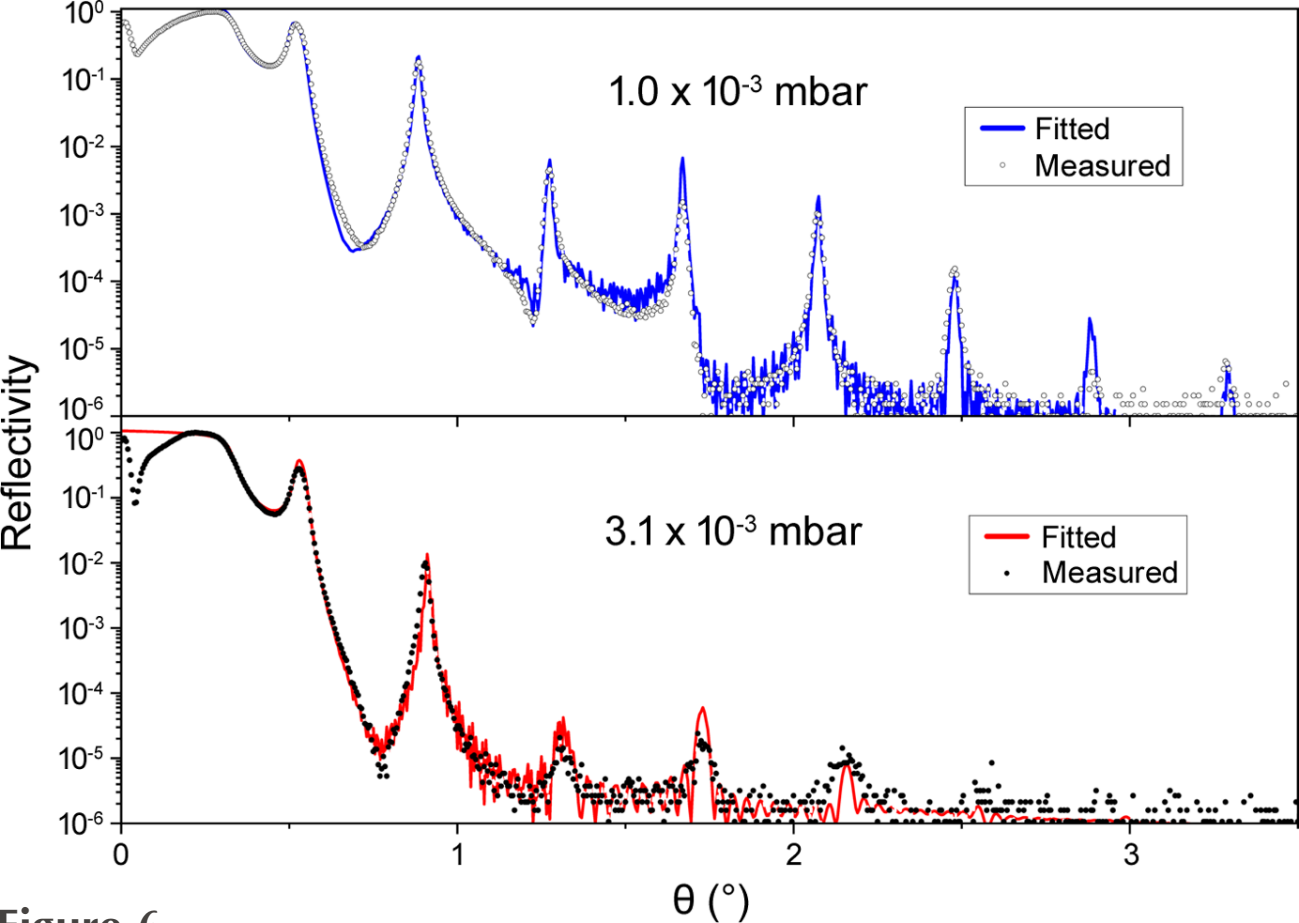
Measured XRR profiles of two Cr/C MLs with two distinct working gas pressures of (top) 1.0 × 10^−3^ mbar and (bottom) 3.1 × 10^−3^ mbar. Both the Cr/C MLs are deposited in dynamic mode.

A series of constant-period Cr/C and W/B_4_C MLs have been fabricated both in static and dynamic modes. Fig. 6 ▸ illustrates two XRR profiles of Cr/C MLs (dimensions *L* = 30 mm × *W* = 25 mm), with each profile corresponding to a distinct working gas pressure utilized during fabrication, conducted in dynamic mode. As expected, the quality of the Cr/C ML has been enhanced by reducing the gas pressure from 3.1 × 10^−3^ mbar to 1.0 × 10^−3^ mbar while maintaining a fixed power supply and substrate-to-target distance for both the targets (Morawe *et al.*, 2022 ▸). The power supply is 100 W for Cr and 400 W for C, the substrate-to-target distances are 94 mm (for Cr) and 75 mm (for C). At a pressure of 3.1 × 10^−3^ mbar, the XRR profile indicates that the Bragg peaks following the second peak nearly disappear. However, at 1.0 × 10^−3^ mbar, the six Bragg peaks become more prominent and sharper. The structural parameters fitted comprise a periodicity of 11.13 nm, a Cr layer thickness of 7.86 nm, a C layer thickness of 3.27 nm and a total number of layer pairs (*N*) amounting to 34. The sample fabricated at 1.0 × 10^−3^ mbar exhibits an average interlayer diffuseness and roughness of approximately 0.5 nm. Increasing the working pressure to 3.1 × 10^−3^ mbar results in a higher roughness value of up to 1.5 nm. The deposition rates are 0.25 nm s^−1^ for Cr and 0.12 nm s^−1^ for C. The degradation of film quality at higher sputtering pressures is due to the increased collisions and scattering of sputtered atoms and reduced kinetic energy and mobility of the atoms adsorbed on the substrate surface (Zhou *et al.*, 2010 ▸).

**Figure 7:**
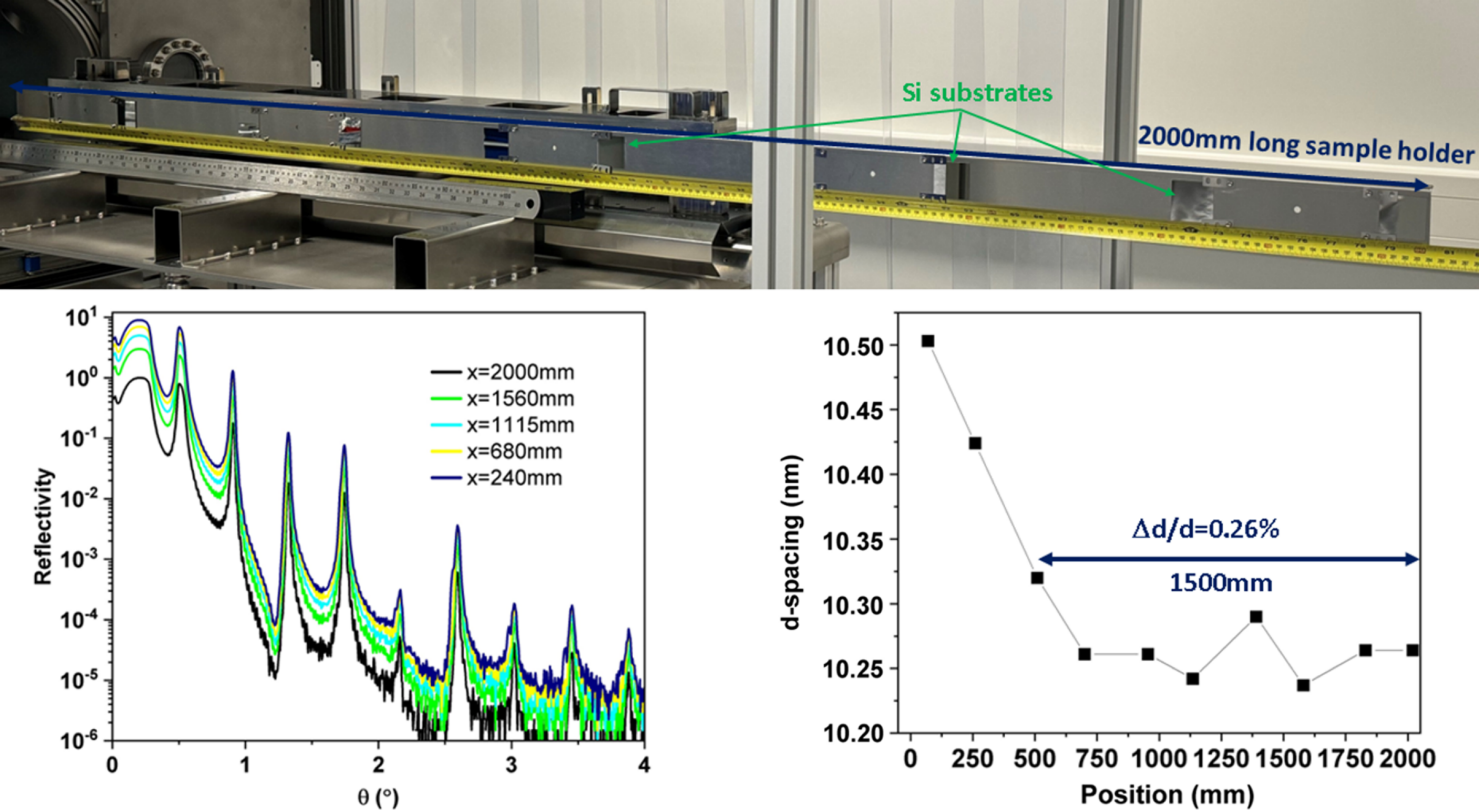
(Top) Photograph of a 2000 mm-long sample holder with Si substrates mounted on top. (Bottom left) Measured XRR profile of the periodic 20-layer pair Cr/C ML fabricated in dynamic mode. (Bottom right) Periodic thickness (*d*) variation as a function of length over 2000 mm.

To test the feasibility of depositing an ML on a longer substrate, ten pieces of Si substrates with the dimensions 40 mm × 40 mm have been mounted on 2000 mm-long sample holder. Due to the limited length of the interlock chamber, the long sample must be transferred directly into the deposition chamber. A constant period Cr/C ML with *N* = 20 has been fabricated in dynamic mode with the following deposition parameters: a working argon gas pressure of 1.0 × 10^−3^ mbar, substrate-to-target distance of 81 mm, and cathode power of 100 W for Cr and 400 W for C. In Fig. 7 ▸, the measured XRR profile of a Cr/C ML with period uniformity over 2000 mm along the length of the ML of motion is shown. The fitted results show that the periodicity remains fixed at 10.3 nm (*d*_Cr_ = 4.1 nm and *d*_C_ = 6.2 nm, with an error of ±0.088 nm, and the corresponding thickness uniformity Δ*d*/*d* is 0.8% over the 2000 mm region). The results indicate the motion speed on one side of the sample is not as uniform as the rest of the samples. Further optimization of motion parameters will be required to improve the thickness uniformity. If we only consider the rest of 1500 mm region, the thickness uniformity is about Δ*d*/*d* = 0.26%. The fitting roughness values of the Cr layer and C layer are 0.48 nm and 0.40 nm, respectively.

**Figure 8:**
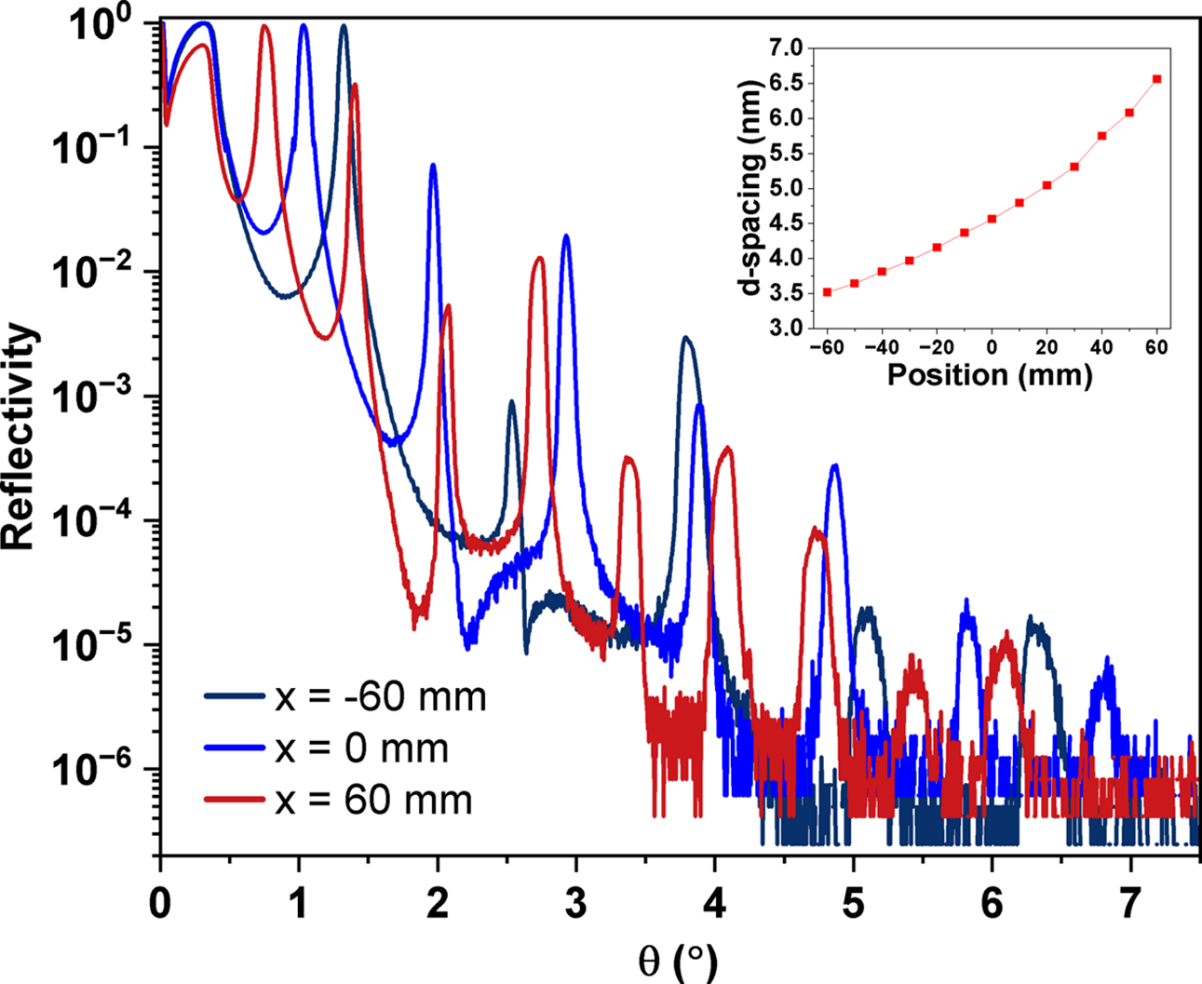
XRR profile of lateral-graded W/B_4_C ML over 120 mm length at three positions: 0 mm (middle) and (two edge) −60 mm and 60 mm. The period thickness varied from 3.52 nm to 6.56 nm. The inset of the figure indicates the variation of the period.

Following the fabrication of constant-period MLs, lateral-graded W/B_4_C MLs are also fabricated using dynamic mode with the following deposition parameters: working argon gas pressure of 3.2 × 10^−3^ mbar, substrate-to-target distance of 81 mm, and a cathode power of 100 W for W and 400 W for B_4_C. The ML is fabricated on an Si substrate of the dimensions 140 mm × 25 mm. In Fig. 8 ▸, the measured XRR profile of a W/B_4_C lateral-graded ML is fabricated with a varying period of 3.52–6.56 nm with a fixed number of layer pairs, *N* = 40. The fitted results indicate a roughness of approximately 0.3 nm, and the densities of the W and B_4_C layers are 18.4 g cm^−3^ (95% of bulk density) and 2.52 g cm^−3^ (100% of bulk density), respectively. Note that we only show this as an example of dynamic mode with a higher gas pressure, and better thin-film quality will be expected if the gas pressure is reduced to 1.0 × 10^−3^ mbar. Further commissioning will be carried out to optimize the speed profile parameter to meet the specifications of the ML for polarization applications.

## 5. Summary

A state-of-the-art MDS has been designed, constructed and successfully installed at DLS. Initial commissioning results validate that the system has met all technical specifications. The ongoing commissioning phase aims to enhance the uniformity along the sagittal direction and further optimize various deposition parameters to improve the overall performance of the MDS. We anticipate that the refined system will produce high-quality X-ray ML optics across diverse applications. These advancements are crucial not only for the enhancement of current beamline capabilities, but also for the anticipated Diamond-II upgrade project.

## Figures and Tables

**Table 1:** Major functional specification of the MDS

Item	Specification
Maximum sample size	1000 mm (long) × 100 mm (wide) × 100 mm (thick) (with inter-lock to load sample)
	2000 mm (long) × 100 mm (wide) × 100 mm (thick) (load sample directly into the deposition chamber)
Cathodes	Eight rectangular 254 mm × 89 mm
Target-to-substrate distance	Manual motion (50–100 mm)
Fast shutter for each target	Time for closing and opening <1 s
Bake-out system	<150°C
Carrier speed range	0.1–100 mm s^−1^
Carrier speed accuracy at 10–50 mm s^−1^	±0.1%
Carrier travel range	4200 mm
Sputtering chamber base pressure	<5×10^−8^ mbar
Load lock chamber sample treatment	Annealing (max 700°C) + UV lamp
DC sputter source stability	±0.1% over 24 h
Process gas control capability	Upstream and downstream
Process gas pressure stability	±0.5% over 24 h

## Data Availability

The authors confirm that the data supporting the findings of this study are available within the article and its supplementary materials.
